# Anxiety and Depression in Drug-Dependent Patients with Cluster C Personality Disorders

**DOI:** 10.3389/fpsyt.2018.00019

**Published:** 2018-02-08

**Authors:** Carlos Roncero, Adelia de Miguel, Ascensión Fumero, Alfonso C. Abad, Rita Martín, Juan Manuel Bethencourt, Lara Grau-López, Laia Rodríguez-Cintas, Constanza Daigre

**Affiliations:** ^1^Psychiatric Service, University of Salamanca Health Care Complex, Institute of Biomedicine of Salamanca, University of Salamanca, Salamanca, Spain; ^2^Addiction and Dual Diagnosis Unit, Hospital Universitari Vall d’Hebron-ASPB, Barcelona, Spain; ^3^Psychiatry Department, Universitat Autònoma de Barcelona, Barcelona, Spain; ^4^Sciences of Health School, Universidad of La Laguna, Tenerife, Spain; ^5^Psychiatry Services, Hospital Universitario Vall d’Hebron, CIBERSAM, Barcelona, Spain

**Keywords:** addiction, substance-use disorder, personality, cluster C, anxiety, depression

## Abstract

**Objective:**

Comorbidity between personality disorders (PD) and substance-use disorders (SUD) is one of the most common findings in the psychiatric field. The patients with Cluster C disorders present maladjustment traits often characterized by high levels of anxiety. The main aim of this study was to find evidences about higher anxiety and depression prevalence on Cluster C than others Clusters, analyzing similarities and differences within, with other Cluster A and B PD patients and patients without PD.

**Method:**

A total of 822 substance dependent patients (ages18–78; Mean = 38.35, SD = 10.14) completed the structured clinical interview for DSM-IV Axis I and Axis II disorders, Beck Depression Inventory, and State-Trait Anxiety Inventory.

**Results:**

Results supported poly-consumption in Cluster C patients, being greater alcohol consumption as well as abuse of both stimulants and depressants. Anxiety and depression did not show just one pattern for all patients with SUD-Cluster C PD. There was a relation between anxiety and depression for all the groups except for the Dependent-PD.

**Conclusion:**

Interventions should focus on aspects like depression and anxiety more than on the substance consumed.

## Introduction

Personality disorders (PDs) are behavioral patterns with rigid traits that remain stable over time. The individual’s developmental stage or social–cultural environment should not be used as an explanation of impairments in personality functioning and the individual’s personality trait expression as it is explained in DSM-5. They directly affect perceptions, the way of thinking and relating to the world, and the control of individual impulses (1). Comorbidity of Axis I disorders complicates both the diagnosis and prognosis of PD (2). One of the most common comorbidities of patients with PD involves substance-use disorders (SUDs), and, *vice versa*, maladjustment (3, 4) and PD are more frequent among addicts (5–7).

Between 65 and 90% of individuals with addictions have PD (8, 9). The most commonly detected PD are Limit and Antisocial Cluster B ones; followed by Avoidant, Passive-aggressive and Obsessive–Compulsive PDs from Cluster C; and finally Schizotypal PD from Cluster A (6, 10). However, there are discrepancies, and not all studies coincide with this ranking. These discrepancies could be explained by the type of substance being consumed; the assessment methods or techniques used; the time of the assessment; being outpatients or inpatients; poly-consumption (11, 12), or even the stability of the disorders (13).

DSM-IV criteria seem to be a valid tool for the diagnosis of PD. A previous study supported the validity of the DSM-IV criteria for Avoidant PD in Hispanic population with SUDs (14).

Personality disorders from Cluster C (or core anxiety) group together excessively nervous or fearful individuals, who are characterized by great emotional instability that is evidenced by personal passivity and suffering. This cluster consists of Avoidant, Dependent, and Obsessive–Compulsive PDs (1). Cluster C traits could be detected even in adolescent population (15). Adolescents with maladaptative personality traits are more at risk of developing cannabis dependence or other SUDs (16).

To explain the association between PD Cluster C and SUD, two hypotheses have been put forward. One is the self-medication hypothesis, which proposes that individuals discover that substances alleviate or change a series of painful affective states (17, 18). In the specific case of Cluster C, directly linked to anxiety, it is hypothesized that consumption of substances is aimed at counteracting feelings of isolation and a lack of social relations. Consequently, drugs can be used to substitute these relations or simply produce a feeling of tranquility in social situations. These characteristics indicate a tendency among patients in this group of disorders toward an overall greater drug consumption, including alcohol and other substances, both stimulants and depressants (19, 20).

The second hypothesis, known as *stress response dampening*, suggests that individuals with high scores in personality traits like stress reactivity, sensitivity to anxiety, and neuroticism are vulnerable to stressful events. These individuals tend to respond to stress with anxiety and mood swings, which can, in turn, push them toward substance consumption (2).

Beyond these hypotheses, previous studies have shown anatomo-functional correlates between Cluster C addicted patients and their willingness to change (21).

Furthermore, it has been suggested that patients with SUD who have PD usually show more psychopathological problems, mainly anxiety and depression (8, 22–25). Kranzler et al. (26) highlight that anxiety and depression are substantially higher among SUD patients with PDs than among those without. Additionally, Langås et al. point out that symptoms of depression and addiction are more serious in SUD patients with PD (8). Despite studies on the existence of psychiatric comorbidity between Cluster C and SUD (8, 20) and of the possible influence of anxiety and depression on PD, there have been no studies involving large samples on the influence of anxiety and depression on patients with Cluster C PD.

Therefore, the aim of this work is to determine the prevalence of Cluster C PD in patients with SUD who seek treatment, to analyze differences in clinical variables between the various disorder subtypes of Cluster C and evaluate if the variables of anxiety and depression differentiate these patients from those who do not present disorders of this cluster. We hypothesize that Cluster C disorders will be common in addicts, that there will be clinical and consumption differences among the different disorders of this cluster, and that anxiety and depression will be distinct in each disorder and will influence clinical variables differently.

## Materials and Methods

### Participants

This descriptive study involved a clinical sample of 822 substance dependent patients according to the DSM-IV-TR criteria (621 males and 201 females, mean age = 38.35, SD = 10.14, range 18–78). The study was conducted in the outpatient drug clinic of Vall d’Hebron Hospital’s Psychiatry Service of the (CAS Vall d’Hebron) in Barcelona, Spain.

### Measures

Patients were visited by a psychiatrist, and three psychological evaluation visits were carried out by psychologists. The psychological evaluation visits were carried out in the first month of starting treatment in our Unit. Sociodemographic and clinical data were recorded using a questionnaire designed *ad hoc*, as well as evaluating all patients using the following instruments.

SCID I (The Structured Clinical Interview for DSM-IV Axis I disorders), a widely used scale that has shown good psychometric properties, was used to assess SUD (27).

SCID II (The Structured Clinical Interview for DSM-IV for Axis II) was used to assess PDs. This tool has shown adequate reliability and usefulness providing clear discrimination among Axis II disorders (28).

Beck Depression Inventory (BDI) (29, 30) is scored by summing the rating for all its 21 items to obtain a total score ranging from 0 to 63. Each symptom’s severity is rated on a 4-point scale (0–3).

State-Trait Anxiety Inventory (STAI) (31) is a 40-item measure that indicates the intensity of feelings of anxiety. It distinguishes between state anxiety (i.e., a temporary condition experienced in specific situations) and trait anxiety (i.e., a general tendency to perceive situations as threatening). Each scale consists of 20 items rated on a 4-point Likert scale. STAI has demonstrated good internal consistency, test–retest reliability in trait anxiety, sensitivity to detection of stress in state anxiety, and convergent and discriminant validity (32–35).

### Procedure

Data were collected between January 2005 and June 2013. Inclusion criteria were patients were 18 years or older, presented substance dependence according to DSM-IV-TR, provided a signed consent form to participate in the study, and finished the assessment process. Exclusion criteria were: intoxication at baseline examination, severe somatic disease at baseline examination, and low language proficiency. The study protocol was approved by the hospital’s ethics committee (Comité Ético de Investigación Clínica Vall d’Hebron). Patients did not receive monetary compensation for their participation in the study. This study was part of a wider project on comorbidity on drug dependence.

Using IBM SPSS 19, cross-tabs with χ^2^, differential analysis with Student’s *t* and Cohen’s *d*, and Pearson’s *r* were performed. To study poly-consumption, regardless of the main drug being treated, data were crossed on different substance use and the variable PD criteria. To facilitate calculations, each use/or abuse variable is dichotomized in such a way that no-use is scored with a 0 and the use-abuse-dependence is 1.

## Results

### Demographics and Comorbidity PD-SUD

In the sample, 9.5% of patients fulfilled the criteria for at least one PD from Cluster C, with the most frequent diagnosis being Avoidant PD. In addition, 28.9% fulfilled PD criteria for Clusters A and B but not for C, and 61.6% did not fulfill the criteria for any PD (see Table 1).

**Table 1:** Social, demographical, and clinical characteristics.

				**Without personality disorder**		**Clusters A and B**		**Cluster C**		**Cluster C**							
		**Total**								**Avoidant**		**Depend**		**Obs–comp**		**Comorb.**	
								
		***n***	**%**	***n***	**%**	***n***	**%**	***n***	**%**	***N***	**%**	***n***	**%**	***n***	**%**	***n***	**%**
		
		822	100	506	61.8	238	28.9	78	9.5	35	44.9	9	11.5	29	37.2	5	6.4

Sex	Males	621	75.5	388	76.7	178	74.8	55	70.5	24	68.6	3	33.3	24	85.7	4	75.7
	Females	201	24.5	118	23.3	60	25.2	23	29.5	11	31.4	6	66.7	5	14.3	1	24.3

Age	Mean	38.35		39.85		35.29		37.69		37.91		36.33		38.21		35.6	
	SD	10.12		10.61		8.50		9.07		10.15		11.97		9.44		3.78	
	Range	18–78		18–78		18–69		18–60		18–57		21–60		22–58		32–41	

		*F* = 17.11, *p* = 0.000								*F* = 0.339, *p* = 0.797							

Substance-use disorders-treatment	Alcohol	233	100	177	76.0	34	14.6	22	9.4	10	45.4	1	4.5	11	50.0	0	0.0
	Benzodiazepines	27	100	19	70.3	3	11.1	5	18.5	2	40.0	2	40.0	1	20.0	0	0.0
	Opioids	147	100	75	51.0	63	42.9	9	6.1	4	44.4	0	0.0	4	44.4	1	11.1
	Amphetamines	7	100	4	57.1	3	42.9	0	0.0	0	0.0	0	0.0	0	0.0	0	0.0
	Cocaine	319	100	185	58.0	103	32.3	31	9.7	13	41.9	5	16.1	10	32.3	3	9.7
	Cannabis	89	100	46	51.7	32	36.0	11	12.3	6	54.5	1	9.1	3	27.3	1	9.1

Patients with a Cluster C disorder often had another PD: 14 Avoidant PD, 4 Dependent PD, and 5 Obsessive–Compulsive PD and also met the criteria for one or more PD from Clusters A and B (17.9, 5.12, and 6.4%, respectively within Cluster C). Finally, 5 (6.4%) participants showed two or three PD from Cluster C together with one or more PD from Clusters A and B.

It was found that 70.5% of patients with a Cluster C PD were men, although there were clear differences in the type of PD, Obsessive–Compulsive PD (85.7%) was the most common and to a significantly lesser degree Dependent PD (33.7%) (χ^2^ = 12.19, *p* = 0.007).

The average age of patients was over 35 years old with statistically significant differences between the Without PD group and the group of patients from Clusters A and B (39.85 and 35.29, respectively, *t* = 6.28, *p* < 0.001). Within Cluster C, there were no differences in age (*F* = 0.339, *p* = 0.797).

A different pattern in PD prevalence was identified depending on the main substance consumed. Thus, Dependent PD was mainly present in patients who consumed benzodiazepines, and Obsessive–Compulsive PD were more common among those who consumed alcohol, opioids, cocaine, and cannabis.

### Poly-Consumption of Drugs Regardless of Substance Use of Treatment

Table 2 shows the data for use/abuse of substances depending on their main effect on central nervous system (depressants, stimulants, and hallucinogens).

**Table 2:** Drug abuse of among drug-dependent patients.

									**Cluster C**									
	**Without personality disorder (***n*** = 506)**		**Clusters A and B (***n*** = 238)**		**Cluster C (***n*** = 78)**				**Avoidant (***n*** = 35)**		**Dependent (***n*** = 9)**		**Obsess–com (***n*** = 29)**		**Comorb. (***n*** = 5)**			
	***n***	**%**	***n***	**%**	***n***	**%**	**χ^2^**	***p***	***n***	**%**	***n***	**%**	***n***	**%**	***n***	**%**	**χ^2^**	***p***

**Depressant drugs**																		

Alcohol	431	85.2	190	79.8	57	74.0	7.88	0.02	25	71.4	6	66.7	23	82.1	3	60.0	1.45	0.627
*Only alcohol*	15	2.96	–	–	–	–	–	–	3	8.6	–	–	–	–	–	–	–	–
Benzodiazepine	159	31.4	102	42.9	30	39.0	9.59	0.008	13	37.1	3	33.3	12	42.9	2	40.0	0.42	0.935
*Only benzodiazepines*	1	0.20	–	–	–	–	–	–	1	2.9	1	11.1	–	–	–	–	–	–
Opioid	119	23.5	91	38.2	18	23.4	18.2	0.0001	10	28.6	3	33.3	4	14.3	1	20.0	2.47	0.481
Methadone	103	20.4	78	32.8	15	23.4	11.0	0.004	8	27.4	2	28.4	4	16.4	1	25.0	1.31	0.728

**Stimulant drugs**																		

Amphetamines	92	18.2	59	24.8	20	26.0	5.58	0.06	6	17.7	2	22.2	11	39.3	1	20.0	4.85	0.183
Cocaine	336	66.4	203	85.3	51	66.2	29.6	0.0001	21	60.0	7	77.8	19	67.9	4	80.0	2.33	0.507
*Only cocaine*	2	0.40	–	–	–	–	–	–	2	5.7	1	11.1	–	–	–	–	–	–
Tobacco	405	80.0	197	82.8	45	66.2	21.8	0.0001	20	64.5	5	62.5	18	75.0	2	40.0	1.45	0.693

**Hallucinogen drugs**																		

Ecstasy	99	19.6	59	24.8	12	19.4	2.61	0.271	2	10.3	1	16.7	8	34.8	0	0.0	6.21	0.102
Cannabis	289	57.1	167	70.2	45	58.4	11.6	0.003	17	48.6	6	66.7	18	64.3	4	80.0	4.41	0.221
*Only cannabis*	–	–	1	0.42	–	–	–	–	–	–	1	11.1	–	–	–	–	–	–

Alcohol, tobacco, cocaine, and cannabis were the drugs consumed by the majority of patients. However, patients without PD—compared to those with—showed greater alcohol consumption (χ^2^ = 7.88, *p* = 0.02). Compared to patients with disorders from Cluster A and B, those with Cluster C disorders and those without PD consumed fewer opiates (χ^2^ = 18.2, *p* = 0.000) and less methadone (χ^2^ = 11.0, *p* = 0.004). There was a higher percentage of patients with PD that consumed benzodiazepines (χ^2^ = 9.59, *p* = 0.008) compared to those patients without PD.

In the case of stimulants, patients with Cluster C PD typically showed lower tobacco consumption (χ^2^ = 21.8, *p* < 0.0001), whereas cocaine was used by patients from Clusters A and B (χ^2^ = 29.6, *p* < 0.0001), there were no differences among amphetamine use.

Finally, regarding drugs with hallucinogenic effects, there were no differences in the use of ecstasy, but there were for cannabis with patients with Cluster C PD and those Without PD being the ones that consume the least (χ^2^ = 11.6, *p* = 0.003).

Within Cluster C, there were no statistically significant differences though there was a higher percentage of Obsessive–Compulsive PD who consumed alcohol, benzodiazepines, amphetamines, and tobacco compared with the other two PD from Cluster C.

In the sample, 96.6% of patients were poly-consumers with the exception being the Avoidant PD of whom 80% were poly-consumers (28 participants).

There were 15 patients Without PD and 3 Avoidant PD who only consumed alcohol; there was 1 Without PD, 1 Avoidant PD, and 1 Dependent PD only using benzodiazepines; those only on cocaine were 2 Without PD, 2 Avoidant PD, and 1 Dependent PD; and those only on cannabis were 1 Avoidant PD and 1 Cluster A and B PD. In other words, poly-consumption is almost universal in SUD.

### Depressive Symptomatology and Anxiety

**Figure 1:**
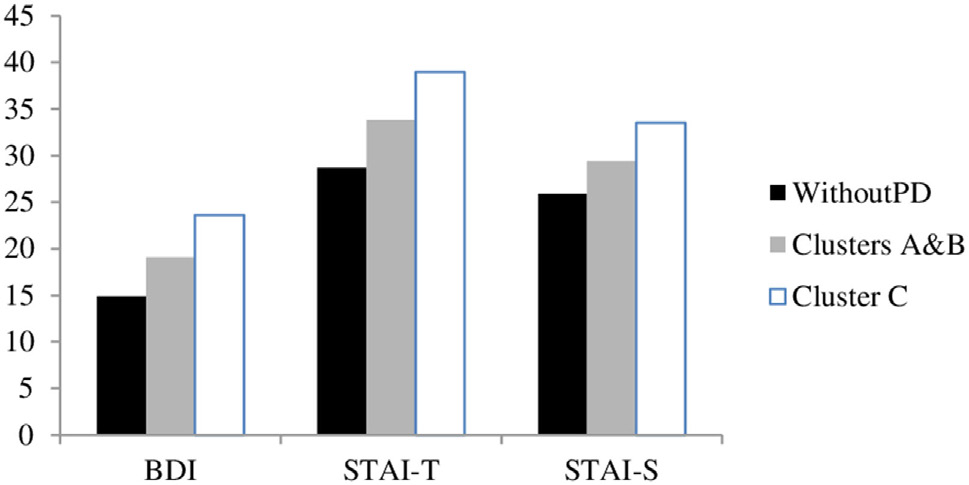
Anxiety and depression according to the instrument of evaluation.

The levels of depression and trait anxiety and state anxiety were studied as a function of Axis II (Figure 1). Significant differences appeared among different clusters and *post hoc* comparisons demonstrated that participants with Cluster C had more depressive symptoms (*F* = 28.63, *p* < 0.001) and greater state anxiety (*F* = 30.60, *p* < 0.001) than those with Cluster A and B PD, and those participants Without PD. However, trait anxiety (*F* = 13.39, *p* < 0.001) was similar for PD within Axis II and higher for participants Without PD.

Having performed comparisons within Cluster C and owing to the size of the sample, it was decided to calculate the size of Cohen’s *d* effect (Table 3). Depressive symptomatology, as measured by BDI, was similar for Avoidant PD and Dependent PD (Cohen’s *d* close to 0), whereas the Obsessive–Compulsive PD scored higher (large Cohen’s *d*).

**Table 3:** Anxiety and depression within Cluster C.

	Avoidant		Dependent		Obsessive–compulsive		Cohen’s *d*		
	M	SD	M	SD	M	SD	Avoidant-Dependent	Avoidant-obsessive	Dependent-Obsessive
BDI	26.3	12.2	27.7	13.7	19.6	9.9	−0.11^a^	0.61^c^	0.69^c^
STAI-T	39.4	11.2	48.1	21.2	35.4	12.2	−0.54^c^	0.34^b^	0.76^c^
STAI-S	34.3	12.9	41.4	21.2	31.3	14.27	−0.42^b^	0.22^b^	0.57^c^

*^a^Close to 0 *d* (*d* ≤ 0.19)*.

*^b^Moderate *d* (0.20 ≤ *d* ≤ 0.50)*.

*^c^Large *d* (*d* > 0.50)*.

*BDI, Beck Depression Inventory; STAI-T and STAI-S, State-Trait Anxiety Inventory*.

Additionally, regarding anxiety there were clear differences within Cluster C: Dependent PD scored higher than Avoidant and Obsessive–Compulsive PD (Cohen’s *d* moderate and large in both comparisons). Those with Avoidant PD scored higher than Obsessive–Compulsive, although the difference was more moderate.

Furthermore, depressive symptomatology and anxiety were significantly positively correlated (Table 4). However, when PD types within Cluster C were analyzed, anxiety was not related to the depressive symptomatology in Dependent PD (*r* = −0.05 and *r* = 0.10 for trait and state anxiety, respectively).

**Table 4:** Relationships among depression and anxiety.

							Cluster C							
	Without personality disorder		Clusters A and B		Cluster C		Avoidant		Dependent		Obsessive–comp.		Comorb.	
	STAI-T	STAI-S	STAI-T	STAI-S	STAI-T	STAI-S	STAI-T	STAI-S	STAI-T	STAI-S	STAI-T	STAI-S	STAI-T	STAI-S

BDI	0.66***	0.62***	0.63***	0.61***	0.53***	0.63***	0.54**	0.80***	-0.05	0.10	0.82***	0.76***	0.96	0.49
STAI-T		0.69***		0.67***		0.77***		0.70***		0.95***		0.77***		0.49

** p ≤ 0.01, *** p ≤ 0.001.

*STAI-T and STAI-S, State-Trait Anxiety Inventory; BDI, Beck Depression Inventory*.

Regardless of having a PD or not, there was a close relationship between depression and anxiety, but it was found that within Cluster C, patients with Dependent PD were different to the rest as their responses did not indicate any relation between depression and anxiety.

### Depressive Symptomatology and Anxiety in Relation to Treatment Indicators

To analyze the relevance of depressive symptomatology and anxiety with objective indicators of treatment, the corresponding correlations between depression, trait anxiety, and state anxiety scales were calculated for three treatment indicators: number of detoxifications, number of admissions and number of treatments in health centers.

In participants Without PD and in participants with Cluster A and B PD, the range of *r* was −0.7 and 0.13, indicating the absence of a relation between psychological factors and treatment indicators. However, for Cluster C, though the general scenario is similar to the one above, correlations of 0.24 appear between the two scales of anxiety and depression. Carrying out a zonal analysis within this cluster (Table 5), in the case of Avoidant PD and Obsessive–Compulsive PD, the correlation pattern approaches 0. However, in Dependent PD, there are high negative correlations between depressive symptomatology and treatment indicators, whereas there are moderately positive ones between trait and state anxiety and the indicators, except for the number of detoxifications. When cases of comorbidity within Cluster C were analyzed, the scenario was different: state anxiety was lower, the greater the number of detoxifications and treatments, while the number of admissions increased with greater depressive symptomatology and greater trait anxiety.

**Table 5:** Relationships among depression/anxiety and treatment keys in Cluster C.

	Avoidant			Dependent			Obsessive–compulsive			Comorb.		
	BDI	STAI-T	STAI-S	BDI	STAI-T	STAI-S	BDI	STAI-T	STAI-S	BDI	STAI-T	STAI-S

Detox	0.06	−0.10	0.03	**−0.64**	0.19	0.00	0.16	0.07	0.18	**0.37**	**0.39**	**−0.50**
Ingress	0.07	0.04	−0.06	−**0.62**	**0.65**	**0.50**	0.10	0.10	0.24	–	–	–
Outpatient T.	−0.07	0.06	v0.09	−0.94*	**0.48**	**0.36**	0.06	0.10	−0.02	−0.10	0.02	**−0.49**

*Detox, number of inpatient detoxification; Ingress, number of ingress in Therapeutical Community; Outpatient T, outpatient treatment, number of outpatient treatment performed before; STAI-T and STAI-S, State-Trait Anxiety Inventory; BDI, Beck Depression Inventory*.

Correlations with absolute values of 0.30 or above are given in bold type (*p ≤ 0.05).

## Discussion

Almost 40% of the patients studied have a PD and nearly 10% have a PD from Cluster C. The findings obtained in seeking treatment patients attended at outpatient clinical units is consistent with other studies with smaller samples (8, 9, 20, 36) and manly carried out in inpatient units (4, 7, 10, 13) or only taking into account one specific substance (12).

It is possible that a large number of results from studies on comorbidity in SUD could be attributed to the characteristics of the personality pathologies, themselves (37). However, it has also been postulated that some interactions between drugs, like alcohol and nicotine (38) or psychoactive substances used simultaneously, like cocaine and alcohol (39), could explain a certain degree of comorbidity.

The general trend in clinical practice seems to identify anxiety and depression mainly in Cluster B patients and *vice versa* because they seem to present themselves and their symptoms in a more “visible” way. Based on the results of this study it seems to be advisable to identify Cluster C patients in the Addiction Treatment Centers and take into account the fact that this population has high rates of anxiety and depression symptoms.

It is worth noting that patients with Cluster C disorders present maladjustment traits often characterized by high levels of anxiety. Based on the theory of self-medication, SUD could have developed or have been maintained in order to mitigate the consequences of these personality traits. Thus, dealing with these traits and symptoms could be of great interest. Moreover, chronic substance dependence can lead to affective dysregulation, which can contribute to the personality pathology (40). There are new evidence regarding changes in neurobiology and behavior following intermittent ethanol consumption, leading to increase in social anxiety, altered synapses, and reduced neurogenesis, cholinergic, and serotonergic neurons (41).

In Cluster C, PD the most commonly substances for consulting are benzodiazepines. These findings could be explained by the anxiolytic effect of these drugs. On the other hand, patients with PD from this cluster frequently show alcohol dependence (74%). These results support the observed tendency in patients from this group of disorders toward greater alcohol consumption as well as abuse of both stimulants and depressants, which coincides with previous studies (19, 20, 42–45). The prevalence of dependence in legal drugs (alcohol and benzodiazepines) is similar to the other PD group. In contrast, the prevalence of dependence to illegal drugs (opiates, cocaine, and cannabis) is similar to the patients without a PD. It could be hypothesized that they are avoiding the illegal circuits because of obsessive and avoiding cognitions/behaviors and the high levels of stress.

Regardless of the main substance of abuse, poly-consumption is the norm in all patients, though to a lesser extent for those with Avoidant PD. This fact highlights a disturbing situation in which it is necessary to clarify the implications of each substance and their interaction when inside the body. Poly-consumption is an extremely risky practice and requires the assessment of each individual’s particular circumstances to design an appropriate intervention program for that individual’s needs.

The prevalence of men in Cluster C PD is higher, except in the Dependent group, and as opposed to other disorders, there is no correlation between anxiety and depression.

Psychological functioning related to depression and anxiety is characteristic of Cluster C. In fact, the data in this study confirm that patients from Cluster C score higher than other patients (both Without PD and Cluster A and B groups) in depression and state anxiety. However, in their trait anxiety, there are no differences with other PD patients, though they do score higher than patients Without PD. Specifically, within Cluster C, Obsessive–Compulsive PD patients present fewer depressive symptoms and less anxiety (trait and state) than Avoidant and Dependent PD. Therefore, the results on anxiety and depression do not show just one pattern for all the patients with SUD-PD. It is worth highlighting that, as observed in previous studies (46, 47), patients with Cluster C PD are most commonly affected by depression; however, they are not the group with greatest trait anxiety.

Nevertheless, it cannot be ignored that among patients with mood disorders or anxiety, Obsessive–Compulsive, and Avoidant disorders from Cluster C are the most frequent and have the worst prognosis (48, 49). Moreover, the close relation between anxiety and depression for all the groups except for the Dependent PD group clearly shows the complexity of the relation and suggests that in many patients intervention should focus on aspects like depression and anxiety more than on the substance being consumed.

Furthermore, it is important to underline the singularity of the Dependent PD group. For these patients, the negative relation between depression and treatment indicators and positive relation between anxiety and treatment indicators clearly shows that this group within Cluster C should be treated as a distinct group with specific features that need to be considered at treatment level.

This is the first description of the relationship between Cluster C PD and SUD in a clinically large sample. One of the strong points of this study is the use of a systematic evaluation process using semi-structured interviews that provide high diagnostic reliability. As we noted before, the size of most samples studied about these topic have been smaller in comparison to the number of participants included in this research.

The results of the present study should be interpreted in light of the following limitations. The results are limited to patients with PD seeking treatment in a drug unit. It would be very interesting to confirm if SUD patients treated in residential centers show the same prevalence of Cluster C PD; if depression and anxiety are also differential psychological indicators within this Cluster; and if there is the same relation with treatment indicators in a residential center. On the other hand, it seems of interest studying other possible variables such as other comorbidities, the age of onset, family history, and so on.

Finally, it is necessary to analyze the possible implications of basic temperamental traits both in the description of SUD—Cluster C patients as well as in the differential treatment of their addiction.

Reciprocal relationship between substance use, mood, and anxiety disorders has been found (49–51). Given the prevalence of Cluster C disorders in addicts, their systematic assessment should be implemented for all patients seeking treatment. The presence of depression symptoms and anxiety traits can be useful to detect the most serious patients and with whom a specific approach should be adopted, especially in Dependent PD.

## Author Contributions

Every author contributed in study design and manuscript preparation, literature review, data collection and analysis, and has approved the final manuscript.

## Conflict of Interest Statement

The authors declare that the research was conducted in the absence of any commercial or financial relationships that could be construed as a potential conflict of interest.

## References

[1] American Psychiatric Association. Diagnostic and Statistical Manual of Mental Disorders: DSM-IV-TR. 4th ed Washington, DC: American Psychiatric Association (2000). text rev.

[2] Verheul R. Co-morbidity of personality disorders in individuals with substance use disorders. Eur Psychiatry (2001) 16:274–82. 10.1016/S0924-9338(01)00578-8 11514129

[3] Lev-Ran S Imtiaz S Rehm J Le Foll B. Exploring the association between lifetime prevalence of mental illness and transition from substance use to substance use disorders: results from the National Epidemiologic Survey of Alcohol and Related Conditions (NESARC). Am J Addict (2013) 22:93–8. 10.1111/j.1521-0391.2013.00304.x 23414492

[4] Grau-López L Daigre C Grau-López L Rodriguez-Cintas L Egido Á Casas M Administrative prevalence of insomnia and associated clinical features in patients with addiction during active substance use. Actas Esp Psiquiatr (2016) 44(2):64–71. 27099212

[5] Comín M Redondo S Daigre C Grau-López L Casas M Roncero C. Clinical differences between cocaine-dependent patients with and without antisocial personality disorder. Psychiatry Res (2016) 246:587–92. 10.1016/j.psychres.2016.10.083 27839828

[6] Roncero C Daigre C Barral C Ros-Cucurull E Grau-López L Rodríguez-Cintas L Neuroticism associated with cocaine-induced psychosis in cocaine-dependent patients: a cross-sectional observational study. PLoS One (2014) 9:e106111. 10.1371/journal.pone.0106111 25254365 PMC4177812

[7] Thomas VH Melchert TP Banken JA. Substance dependence and personality disorders: comorbidity and treatment outcome in an inpatient treatment population. J Stud Alcohol (1999) 60:271–7. 10.15288/jsa.1999.60.271 10091966

[8] Langås A-M Malt UF Opjordsmoen S. In-depth study of personality disorders in first-admission patients with substance use disorders. BMC Psychiatry (2012) 12:180. 10.1186/1471-244X-12-180 23107025 PMC3514215

[9] Pérez PJE Puerta García C Lagares Roibas A Sáez Maldonado A Prevalencia e intensidad de trastornos de personalidad en adictos a sustancias en tratamiento en un centro de atención a las drogodependencias. Trastor Adict (2003) 5:241–55. 10.1016/S1575-0973(03)70117-2

[10] Skinstad AH Swain A. Comorbidity in a clinical sample of substance abusers. Am J Drug Alcohol Abuse (2001) 27:45–64. 10.1081/ADA-100103118 11373036

[11] Kokkevi A Stefanis N Anastasopoulou E Kostogianni C. Personality disorders in drug abusers: prevalence and their association with AXIS I disorders as predictors of treatment retention. Addict Behav (1998) 23:841–53. 10.1016/S0306-4603(98)00071-9 9801720

[12] López Durán A Becoña Iglesias E. [Patterns and personality disorders in persons with cocaine dependence in treatment]. Psicothema (2006) 18:578–83. 17296090

[13] Vergara-Moragues E González-Saiz F Lozano OM Verdejo García A. Psychopathological stability of personality disorders in substance abuse patients treated in a therapeutic community. J Addict Dis (2013) 32:343–53. 10.1080/10550887.2013.854154 24325768

[14] Becker DF Añez LM Paris M Bedregal L Grilo CM. Factor structure and diagnostic efficiency of the diagnostic and statistical manual of mental disorders, fourth edition, criteria for avoidant personality disorder in Hispanic men and women with substance use disorders. Compr Psychiatry (2009) 50:463–7. 10.1016/j.comppsych.2008.10.002 19683617

[15] Fonseca-Pedrero E Paino M Lemos-Giráldez S Muñiz J Cluster C maladaptative personality traits in a general population of adolescents. Actas Esp Psiquiatr (2013) 41(2):97–105. 23592069

[16] Blecha L Benyamina A Reynaud M [Family management of cannabis in adolescent]. Arch Pediatr (2010) 17(2):191–4. 10.1016/j.arcped.2009.09.018 19892535

[17] Arendt M Rosenberg R Fjordback L Brandholdt J Foldager L Sher L Testing the self-medication hypothesis of depression and aggression in cannabis-dependent subjects. Psychol Med (2007) 37:935–45. 10.1017/S0033291706009688 17202003

[18] Khantzian EJ. The self-medication hypothesis of substance use disorders. A reconsideration and recent applications. Harv Rev Psychiatry (1997) 4(5):231–44. 10.3109/10673229709030550 9385000

[19] Benito A Haro G Orengo T González M Fornés T Mateu C [Opiate dependence type II or antisocial: Cloninger’s psychobiological model and its usefullness in addictions]. Adicciones (2012) 24:131–8. 10.20882/adicciones.106 22648316

[20] Lorea I Fernández-Montalvo J López-Goñi JJ Landa N [Cocaine addiction and personality disorders: a study with the MCMI-II]. Adicciones (2009) 21:57–63. 10.20882/adicciones.252 19333525

[21] Moreno-López L Albein-Urios N Martínez-González JM Soriano-Mas C Verdejo-García A. Prefrontal gray matter and motivation for treatment in cocaine-dependent individuals with and without personality disorders. Front Psychiatry (2014) 5:52. 10.3389/fpsyt.2014.00052 24904436 PMC4032993

[22] Van Horn DH Frank AF. Substance-use situations and abstinence predictions in substance abusers with and without personality disorders. Am J Drug Alcohol Abuse (1998) 24:395–404. 10.3109/00952999809016905 9741942

[23] Nace EP Davis CW Gaspari JP. Axis II comorbidity in substance abusers. Am J Psychiatry (1991) 148:118–20. 10.1176/ajp.148.1.118 1984695

[24] Agrawal A Narayanan G Oltmanns TF. Personality pathology and alcohol dependence at midlife in a community sample. Personal Disord (2013) 4(1):55–61. 10.1037/a0030224 23230852 PMC3575957

[25] Hatzigiakoumis DS Martinotti G Giannantonio MD Janiri L. Anhedonia and substance dependence: clinical correlates and treatment options. Front Psychiatry (2011) 2:10. 10.3389/fpsyt.2011.00010 21556280 PMC3089992

[26] Kranzler HR Satel S Apter A. Personality disorders and associated features in cocaine-dependent inpatients. Compr Psychiatry (1994) 35:335–40. 10.1016/0010-440X(94)90272-0 7995024

[27] First MB Spitzer RL Gibbon M Williams JBW Structured Clinical Interview for DSM-IV^®^ Axis I Disorders (SCID-I), Clinician Version, Administration Booklet. New York: American Psychiatric Publication (2012).

[28] First MB User’s Guide for the Structured Clinical Interview for DSM-IV Axis II Personality Disorders: SCID-II. New York: American Psychiatric Publication (1997).

[29] Barral C Rodríguez-Cintas L Martínez-Luna N Bachiller D Perez-Pazos J Alvarós J Reliability of the beck depression inventory in opiate dependent patients. J Subst Use (2016) 21(2):128–32. 10.3109/14659891.2014.980859

[30] Beck AT Ward CH Mendelson M Mock J Erbaugh J An inventory for measuring depression. Arch Gen Psychiatry (1961) 4:561–71. 10.1001/archpsyc.1961.01710120031004 13688369

[31] Spielberger CD Gorsuch RL Lushene RE The State-Trait Anxiety Inventory. Preliminary Test Manual for Form X. Tallahassee: Florida State University (1970). [Spanish adaptation, Cuestionario de Ansiedad Estado-Rasgo. Manual, 1986, Madrid: Tea Ediciones].

[32] Barnes LLB Harp D Jung WS Reliability generalization of scores on the Spielberger State-Trait Anxiety Inventory. Educ Psychol Meas (2002) 62:603–18. 10.1177/0013164402062004005

[33] Hishinuma ES Miyamoto RH Nishimura ST Nahulu LB Andrade NN Makini GK Psychometric properties of the State-Trait Anxiety Inventory for Asian/Pacific-islander adolescents. Assessment (2000) 7:17–36. 10.1177/107319110000700102 10668003

[34] Kabacoff RI Segal DL Hersen M Van Hasselt VB. Psychometric properties and diagnostic utility of the Beck Anxiety Inventory and the State-Trait Anxiety Inventory with older adult psychiatric outpatients. J Anxiety Disord (1997) 11:33–47. 10.1016/S0887-6185(96)00033-3 9131880

[35] Vautier S. A longitudinal SEM approach to STAI data: two comprehensive multitrait-multistate models. J Pers Assess (2004) 83:167–79. 10.1207/s15327752jpa8302_11 15456653

[36] Lysaker PH Olesek K Buck K Leonhardt BL Vohs J Ringer J Metacognitive mastery moderates the relationship of alexithymia with cluster C personality disorder traits in adults with substance use disorders. Addict Behav (2014) 39(3):558–61. 10.1016/j.addbeh.2013.11.007 24300836

[37] Jahng S Trull TJ Wood PK Tragesser SL Tomko R Grant JD Distinguishing general and specific personality disorder features and implications for substance dependence comorbidity. J Abnorm Psychol (2011) 120:656–69. 10.1037/a0023539 21604829 PMC4241053

[38] Funk D Marinelli PW Lê AD. Biological processes underlying co-use of alcohol and nicotine: neuronal mechanisms, cross-tolerance, and genetic factors. Alcohol Res Health (2006) 29:186–92. 17373407 PMC6527043

[39] McCance EF Price LH Kosten TR Jatlow PI. Cocaethylene: pharmacology, physiology and behavioral effects in humans. J Pharmacol Exp Ther (1995) 274:215–23. 7616402

[40] Sher KJ Grekin ER Alcohol and affect regulation. In: Gross JJ, editor. Handbook of Emotion Regulation. New York: Guilford Press (2007). p. 560–80.

[41] Crews FT Vetreno RP Broadwater MA Robinson DL. Adolescent alcohol exposure persistently impacts adult neurobiology and behavior. Pharmacol Rev (2016) 68(4):1074–109. 10.1124/pr.115.012138 27677720 PMC5050442

[42] DeJong CA van den Brink W Harteveld FM van der Wielen EG. Personality disorders in alcoholics and drug addicts. Compr Psychiatry (1993) 34:87–94. 10.1016/0010-440X(93)90052-6 8387417

[43] Echeburúa E de Medina RB Aizpiri J. Alcoholism and personality disorders: an exploratory study. Alcohol Alcohol (2005) 40:323–6. 10.1093/alcalc/agh158 15824064

[44] Harrison ER Haaga J Richards T. Self-reported drug use data: what do they reveal? Am J Drug Alcohol Abuse (1993) 19:423–41. 10.3109/00952999309001632 8273764

[45] Preuss UW Johann M Fehr C Koller G Wodarz N Hesselbrock V Personality disorders in alcohol-dependent individuals: relationship with alcohol dependence severity. Eur Addict Res (2009) 15:188–95. 10.1159/000228929 19622885 PMC2790737

[46] Hardy GE Barkham M Shapiro DA Stiles WB Rees A Reynolds S. Impact of Cluster C personality disorders on outcomes of contrasting brief psychotherapies for depression. J Consult Clin Psychol (1995) 63:997–1004. 10.1037/0022-006X.63.6.997 8543722

[47] Morse JQ Pilkonis PA Houck PR Frank E Reynolds CF. Impact of cluster C personality disorders on outcomes of acute and maintenance treatment in late-life depression. Am J Geriatr Psychiatry (2005) 13:808–14. 10.1097/00019442-200509000-00010 16166411

[48] Grant BF Hasin DS Stinson FS Dawson DA Chou SP Ruan WJ Prevalence, correlates, and disability of personality disorders in the United States: results from the national epidemiologic survey on alcohol and related conditions. J Clin Psychiatry (2004) 65:948–58. 10.4088/JCP.v65n0711 15291684

[49] Grant BF Hasin DS Stinson FS Dawson DA Patricia Chou S June Ruan W Co-occurrence of 12-month mood and anxiety disorders and personality disorders in the US: results from the national epidemiologic survey on alcohol and related conditions. J Psychiatr Res (2005) 39:1–9. 10.1016/j.jpsychires.2004.05.004 15504418

[50] Grant BF Goldstein RB Chou SP Huang B Stinson FS Dawson DA Sociodemographic and psychopathologic predictors of first incidence of DSM-IV substance use, mood and anxiety disorders: results from the Wave 2 National Epidemiologic Survey on alcohol and related conditions. Mol Psychiatry (2009) 14:1051–66. 10.1038/mp.2008.41 18427559 PMC2766434

[51] Dimaggio G D’Urzo M Pasinetti M Salvatore G Lysaker PH Catania D Metacognitive interpersonal therapy for co-occurrent avoidant personality disorder and substance abuse. J Clin Psychol (2015) 71(2):157–66. 10.1002/jclp.22151 25557644

